# The systemic impact of different COVID-19 vaccines in 2-[18F] FDG-PET/CT

**DOI:** 10.1038/s41598-023-49376-8

**Published:** 2023-12-09

**Authors:** Tina Nazerani-Zemann, Birgit Pernthaler, Gerold Schwantzer, Christian Gstettner

**Affiliations:** 1 Division of Nuclear Medicine, Department of Radiology, Medical University of Graz, Auenbruggerplatz 9A, 8038 Graz, Austria; 2 Institute for Medical Informatics, Statistics and Documentation, Medical University of Graz, Graz, Austria

**Keywords:** Molecular medicine, Respiratory tract diseases, Cancer

## Abstract

Austria started its COVID-19-vaccination program in December 2020 with three different vaccines. As the vaccination program continues, we encountered increased 2-[18F] FDG-activity not only in axillary lymph nodes ipsilateral to the injection site but also in other organs. The aim of this retrospective study is to present results of the metabolic activity of ipsilateral axillary lymph nodes, liver, blood pool, spleen, and bone marrow after three different vaccines. To our knowledge, this is the first study to examine systemic response changes in relation to time after COVID-19 vaccination using three different vaccines. The collected data of 220 eligible vaccinated patients (127 with BioNTech/Pfizer BNT162b2, 61 with Moderna, and 32 with AstraZeneca) examined with 2-[18F] FDG-PET/CT were enrolled. The PET/CT examinations were evaluated from day 1 to day 135 (SD: 23.2, median: 26) after different vaccinations. Seventy-one out of these 220 patients underwent a pre-vaccination 2-[18F] FDG -PET/CT. SUVmax of axillary node(s), and blood pool, liver, spleen, and bone marrow as reference organs were calculated. The ratio of SUVmax activity of axillary lymph node to reference organs was also compared in all patients. The tracer activity dynamics were investigated in three different vaccines. After BioNTech/Pfizer vaccination 2-[18F] FDG activity in axillary lymph nodes shows a steady decrease in all patients. Ten days after vaccination the 2-[18F] FDG uptake was at its highest activity. Seventy days after vaccination, tracer activity is not different from the background activity of 2-[18F] FDG in the axillary region. This result also applies to other two vaccines; however, in the 4th week after Moderna vaccination SUVmax in lymph nodes showed the highest peak of tracer activity. With AstraZeneca the highest activity was at the earlier days. There was no significant statistical difference of SUVmax of lymph nodes or its ratios to other reference organs between three groups of vaccines. SUVmax in lymph nodes was statistically significant lower than SUVmax in the liver, spleen, and bone marrow with *p*-values of < 0.001, 0.044, and 0.001, respectively. In the group of 71 patients with a pre-vaccination PET/CT examination, the median SUVmax of lymph nodes increased significantly after vaccination from 0.82 (IQR 0.59–1.38) to 1.80 (IQR 1.07–3.89)(*p* < 0.001). In contrast median tracer activity in the liver decreased from 3.37 (IQR 2.83–3.91) to 3.11 (2.56–3.70) (*p* = 0.032). There was no significant change of tracer activity after vaccination in other reference regions (mediastinum, spleen, and bone marrow). In this group of 71 patients, there was also no significant difference in tracer activity in different types of vaccines. Local site and ipsilateral axillary lymph node activity in 2-[18F] FDG PET/CT after COVID19-vaccination is suggested in many studies. The main challenge is recognizing the changes in lymph nodes during time after vaccination to minimize false interpretation, foremost in patients with oncological diagnoses. Moreover, different vaccines cause different system metabolic changes. The knowledge of vaccine type, the time interval between vaccination and PET/CT scan is essential, especially in therapy evaluation.

## Introduction

The emergence of the novel SARS-CoV-2 virus in December 2019 has led to the ongoing global coronavirus pandemic. Since as early as January 2020, joint efforts have been made to develop an effective vaccine to control the spread of disease.

More than a hundred different vaccines have been developed using different technologies, many still in clinical trials. The first vaccines developed and authorized by U.S. Food and Drug Administration (FDA) were based on mRNA sequence encoding segments as in BioNTech/Pfizer^1^ and Moderna^2–4^. The ChAdOx1 nCoV-19 vaccine (AstraZeneca) was developed at Oxford University and later approved and authorized in the E.U. based on replication-deficient chimpanzee adenoviral vector ChAdOx1, containing the SARS-CoV-2 structural surface glycoprotein antigen (spike protein; nCoV-19) gene^5^.

Austria started mass vaccination using three different types of vaccines (BioNTech/Pfizer, Moderna, and AstraZeneca) following the SARS-CoV-19 vaccine rollout in Europe. The first vaccine against the coronavirus in Austria was administered on December 27th, 2020, to an elderly volunteer with the BioNTech/Pfizer vaccine^6^. As the vaccination program continues, we have encountered increased F18-fluorodeoxyglucose (2-[18F] FDG) uptake in the axillary lymph nodes, ipsilateral to the injection site, as well as mild accumulation of tracer in the injection site. Due to its not specific uptake character, 2-[18F] FDG accumulates in cells with increased glucose metabolism, for example, tumor cells or inflammation. Transient 2-[18F] FDG accumulation was reported in several studies with other vaccines for influenza, human papillomavirus (HPV), and more^7,8^.

Axillary swelling or tenderness was reported as an adverse event in clinical trials after vaccination with Moderna in 11.6% of recipients after the first dose and 16.0% after the second dose^9^. In BioNTech/Pfizer trials, this was not addressed as an adverse event; however, lymphadenopathy was detected in even more recipients versus a placebo group^10^.

In a recent systematic review, lymphadenopathy caused by COVID-19 vaccination was reported for BioNTech/Pfizer in 44.1%, Moderna in 25%, and Oxford-AstraZeneca in 1.5%, respectively. Recent studies and case reports also suggest the ipsilateral—mostly axillary—lymphadenopathy after vaccination with AstraZeneca^11^. This particular dilemma in the diagnostic field was addressed in many publications, primarily published in radiologic journals, suggesting that vaccine-induced axillary lymphadenopathy may mimic metastasis in oncologic patients^12^. There are also recent reports supporting systematic inflammatory response due to COVID-19 vaccination^13,14^.

This retrospective study presents results of metabolic activity changes in three different vaccines in ipsilateral axillary lymph nodes. We also represent our data regarding tracer activity in the liver, the blood pool, bone marrow and spleen activation after vaccination. The aim of this study is to show any systemic metabolic changes after vaccination with three different vaccines in relation to time. Furthermore, we want to show how these changes could influence the Positron Emission Tomography/Computerised Tomography (PET/CT) examination interpretation. To our knowledge, this is the first study to examine systemic response changes in correlation to time after COVID-19 vaccination using three different vaccines. It is especially of importance in cases of therapy response evaluation.

### Ethics declaration

This retrospective study was performed involving human participation in accordance to relevant guidelines and regulations and with the 1964 Helsinki declaration and its later amendments ethical standards. This study is approved by the regional ethic committee of Medical University of Graz ‘Ethikkommission an der Medizinischen Universität Graz’ with ethic number 33–546 ex 20/21. Informed consent was obtained from the participants.

## Methods

After approval of the local institutional ethic committee (Ethikkommission der Medizinischen Universität Graz), we collected the data of 920 patients aged from 20 to 100 years who were referred to our facility from April 1st, 2021 to May 31st 2021 for routine diagnostics performed with 2-[18F] FDG. The patients were asked for their vaccination program if one existed. Two-hundred-and-forty patients were vaccinated and therefore eligible for our study.

We furthermore collected the primary diagnosis, age at the time of vaccination, relevant past medical history, type of vaccine, number of times vaccinated, site of vaccine injection, and the time interval between vaccination and PET/CT examination and therapy, if one existed. Table 1 summarizes the types and doses of vaccines, diagnoses, and therapies at the time of PET/CT examination.

**Table 1:** Number and percentage of vaccines, diagnoses and therapies at the time of PET/CT examination.

	n	%
Number of vaccine doses		
1	116	52.7
2	104	47.3
Vaccine type		
AstraZeneca	32	14.5
Moderna	61	27.7
BioNTech/Pfizer	127	57.7
Diagnoses		
Bronchus carcinoma	60	27.3
Cervix and ovarian carcinoma	2	1
Gastrointestinaltract carcinoma	23	10.5
Oral and maxillofacial carcinoma	14	5,2
Fever of unknown origin	25	11.4
Hepatocellular carcinoma	5	2.3
Evaluation for liver transplantation	3	1.4
Lymphoma	37	16.8
Breast carcinoma	8	3.6
Melanoma	7	3.2
Myasthenia gravis	1	0.5
Multiple myeloma	8	3.6
Renal cell carcinoma	2	0.9
Pancreas carcinoma	11	5.0
Prostate cancer	1	0.5
Sarcoma	2	0.9
Thyroid carcinoma	1	0.5
Thymus carcinoma	1	0.5
Rheumatological conditions	8	3.7
Urothelial carcinoma	1	0.5
Therapy		
Corticosteroid	4	1.8
Chemotherapy	13	5.9
Chemo- and radiation therapy	6	2.8
Without any therapy	175	79.5
Mestinon	1	0.5
Oral anticoagulation therapy	2	0.9
Operation	12	5.5
Operation and chemotherapy	2	0.9
Operation, chemo- and radiation therapy	2	1
Operation and radiation therapy	2	0.9
Radiation therapy	1	0.5

2-[18F] FDG PET/CT studies were acquired on two PET/CT Scanners (Discovery MI, G.E. Healthcare, Milwaukee, WI, USA). All patients were fasting a minimum of 4–6 h prior to scanning, and the measured blood sugar was within the accepted range according to the EANM guidelines. F18-FDG was administered weight and height adapted (3 MBq/kg body weight up to a maximum dose of 285 MBq = 7.7 mCi) one hour prior to image acquisition according to the EANM dosage chart^15^. Whole-body or skull-base-to-thigh PET images were acquired according to the standard protocol of our division (discontinuously 2 min per bed position). Diagnostic CT scans for attenuation correction were acquired using helical mode with 20 slices without a contrast agent. Both PET and CT scans were reconstructed with a slice thickness of 3.75 mm. Ipsilateral lymph node activity was calculated using the maximum standard uptake value using a predefined spheric volume of interest (VOI) of 4 cm^3^. The maximal standardized uptake value (SUVmax) of lymph nodes, mediastinum (aortic arch), and liver (the fifth segment) was calculated according to the following formula:

SUVmax = 2-[18F] FDG uptake in region of interest (kBq/mL)/injected activity (MBq)/patient lean body weight (kg).

SUVmax was chosen due to its significantly improved reproducibility instead of SUVmean and SUVpeak.

Three experienced nuclear medicine physicians with 3–10 years of experience interpreted all 2-[18F] FDG PET/CT images.

All axillary lymph nodes ipsilateral to the injection sites were observed independently from the primary diagnosis. Tracer activity was calculated in all vaccinated patients. In the case of hypermetabolism in more than one lymph node SUVmax with the highest uptake was measured. Patients with existing disseminated lymph node involvement, especially in the upper diaphragm's region, were excluded. Furthermore, all the patients with pathological tracer activity such as metastases in mediastinum, liver, bone marrow (fourth lumbar vertebral body), and spleen as reference regions were excluded.

2-[18F] FDG uptake intensity was measured alone and in relation to tracer activity were measured similarly in all patients in all reference regions (aortic arc for the mediastinum, segment V in the liver, the fourth lumbar vertebral body in the bone marrow and in the spleen the upper spleen).

Seventy-one (71/220) patients had undergone at least one other 2-[18F] FDG-PET/CT examination before vaccination. In the pre-vaccination 2-[18F] FDG -PET/CT study, SUVmax of the same regions were assessed. We measured tracer activity in the same one or more axillary lymph node(s) ipsilateral to the vaccination site before and after vaccination. Furthermore, activity in mediastinum, liver, spleen, and bone marrow and their relation to axillary tracer activity was assessed.

### Statistical analysis

All categorical variables are reported as frequency and percentage. Continuous variables are reported as mean with standard deviation (SD) and median with the interquartile range (25th percentile–75th percentile). Between group differences in categorical parameters were assessed with the Chi-squared test, continuous parameters with the Mann–Whitney-U test if two groups were compared or the Kruskal–Wallis tests for the three different vaccines. Within group differences for categorical parameters were assessed with the McNemar test, for continuous parameters we used the Wilcoxon signed-rank test. Correlations between parameters were assessed with the Spearman correlation coefficient. All statistical tests were performed using IBM SPSS Statistics Version 27 (Release 27.0.1.0 2020. Armonk (NY), USA: International Business Machines Corporation). A *p*-value < 0.05 was considered statistically significant.

### Informed consent

Informed consent was obtained from the participants and local institutional ethics committee (Ethikkommission der Medizinischen Universität Graz) for the procedure and data and images.

## Results

### Results of all patients after vaccination

Out of a total of 240 vaccinated patients, 220 patients (with mean age of 67.23 and SD of 11.95) were eligible after the exclusion of the above-mentioned criteria (disseminated lymph node involvement or with pathologies in reference regions) for our study. One-hundred-twenty-seven (57.7%) Patients were vaccinated with BioNTech/Pfizer, 61 (27.7%) with Moderna, and 32 (14.5%) with AstraZeneca. PET/CT examinations were evaluated from day 1 to day 135 (mean 31.5, SD 23.2, median 26, IQR 15–42) after different vaccinations.

SUVmax of ipsilateral lymph node activity was slightly higher (mean 3.00, SD 2.75, median 1.83, IQR 1.07–4.15) in Moderna than the other two vaccines (BioNTech/Pfizer: mean 2.69, SD 2.09, median 1.91, IQR 1.19–3.41; AstraZeneca: mean: 2.61, SD 2.24, median 1.85, IQR 1.28–3.14). Whereas the SUVmax of reference organs is slightly higher in Moderna than the others two vaccines. See Table 2.

**Table 2:** SUVmax values of the three different vaccines in the examined regions.

	AstraZeneca	Moderna	BioNTech/Pfizer
SUVmax of			
LN			
Mean (SD)	2.61 (2.241)	3.00 (2.752)	2.69 (2.086)
Median (IQR)	1.85 (1.28–3.44)	1.83 (1.07–4.15)	1.91 (1.19–3.41)
Liver			
Mean (SD)	3.11 (0.699)	3.25 (0.773)	3.30 (0.784)
Median (IQR)	3.06 (2.50–3.69)	3.23 (2.81–3.70)	3.20 (2.75–3.78)
Mediastinum			
Mean (SD)	2.49 (0.639)	2.45 (0.477)	2.62 (0.703)
Median (IQR)	2.44 (1.97–2.95)	2.46 (2.17–2.71)	2.43 (2.17–3.03)
Spleen			
Mean (SD)	2.71 (0.814)	2.69 (0.699)	2.73 (0.816)
Median (IQR)	2.58 (2.35–3.29)	2.68 (2.30–3.16)	2.65 (2.30–3.13)
Bone marrow			
Mean (SD)	2.71 (0.772)	3.35 (3.015)	2.95 (0.872)
Median (IQR)	2.71 (2.17–3.06)	2.82 (2.17–3.53)	2.80 (2.31–3.52)
SUVmax ratio of			
LN/Liver			
Mean (SD)	0.86 (0.772)	0.92 (0.740)	0.84 (0.750)
Median (IQR)	0.56 (0.38–1.14)	0.62 (0.37–1.45)	0.65 (0.39–1.08)
LN/mediastinum			
Mean (SD)	1.05 (0.815)	1.25 (1.134)	1.06 (0.840)
Median (IQR)	0.76 (0.49–1.35)	0.80 (0.48–1.73)	0.79 (0.49–1.32)
LN/spleen			
Mean (SD)	0.99 (0.826)	1.13 (1.066)	1.00 (0.746)
Median (IQR)	0.81 (0.46–1.17)	0.73 (0.41–1.71)	0.81 (0.51–1.23)
LN/bone marrow			
Mean (SD)	0.97 (0.746)	1.02 (0.929)	0.96 (0.867)
Median (IQR)	0.74 (0.45–1.10)	0.73 (0.34–1.44)	0.70 (0.43–1.23)

One hundred and sixteen patients (52.7%) received only one dose of vaccine, and the 104 (47.3%) were fully vaccinated at the time of PET/CT examination.

Most of the patients (n = 175; 79.5%) did not receive any therapy related to their diagnosis at the time of examination.

**Figure 1:**
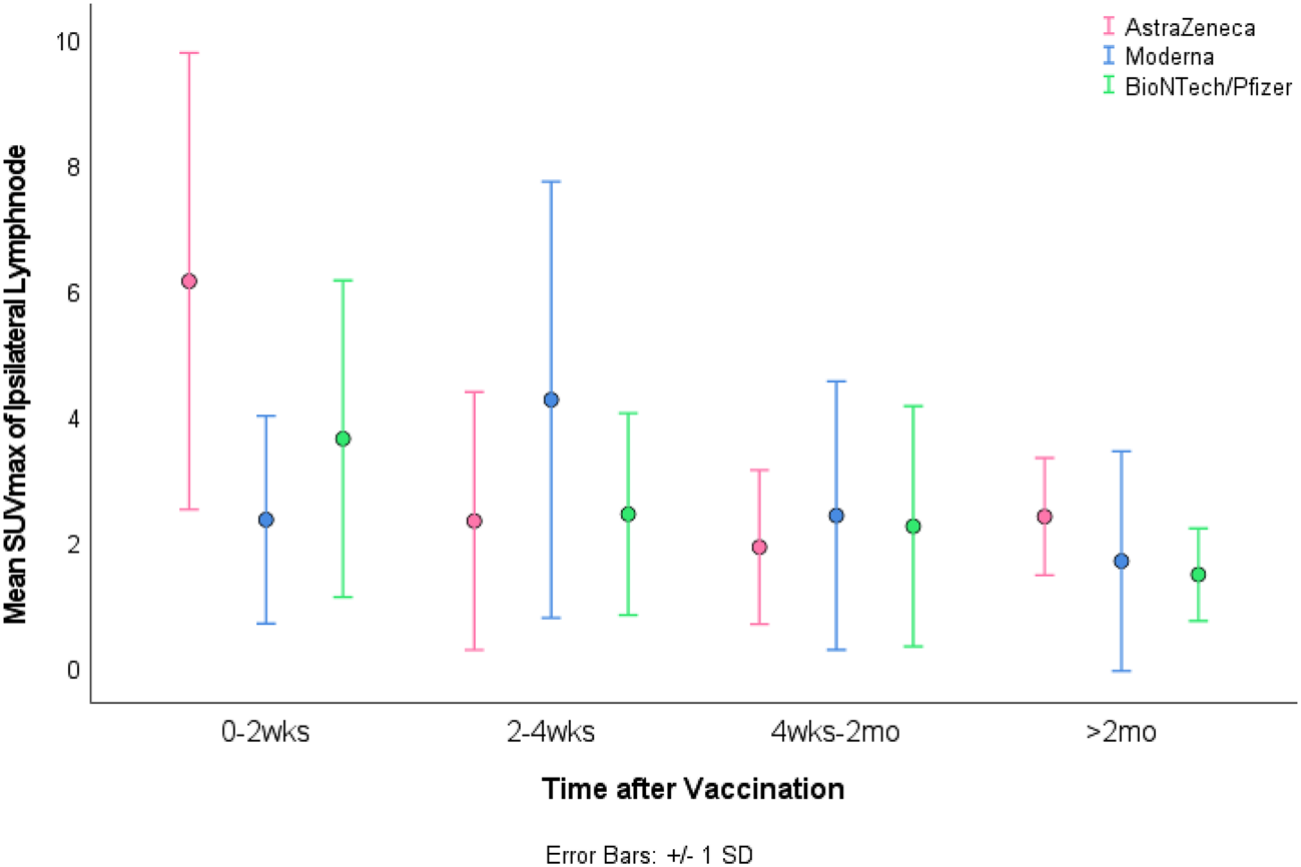
SUVmax in relation to time after vaccination with BioNTech/Pfizer (green), Moderna (blue) and AstraZeneca (red). Mean values represent SUVmax values of days 0–20, 20–40, 40–60 and more than 60 days after vaccination.

After BioNTech/Pfizer the highest 2-[18F] FDG metabolism in axillary lymph nodes was at the 10th day after vaccination with a steady decrease afterwards. Almost more than 70 days after vaccination, tracer activity is not different from the background activity of FDG in the axillary region. The peak of tracer activity in Moderna was in the 4th week after vaccination with a steady decrease afterward. In AstraZeneca no peak of activity was seen but higher values at the earlier days (Fig. 1).

There was no significant statistical difference of SUVmax of lymph nodes or its ratios to other reference organs between the three groups of vaccines (Table 2).

**Figure 2:**
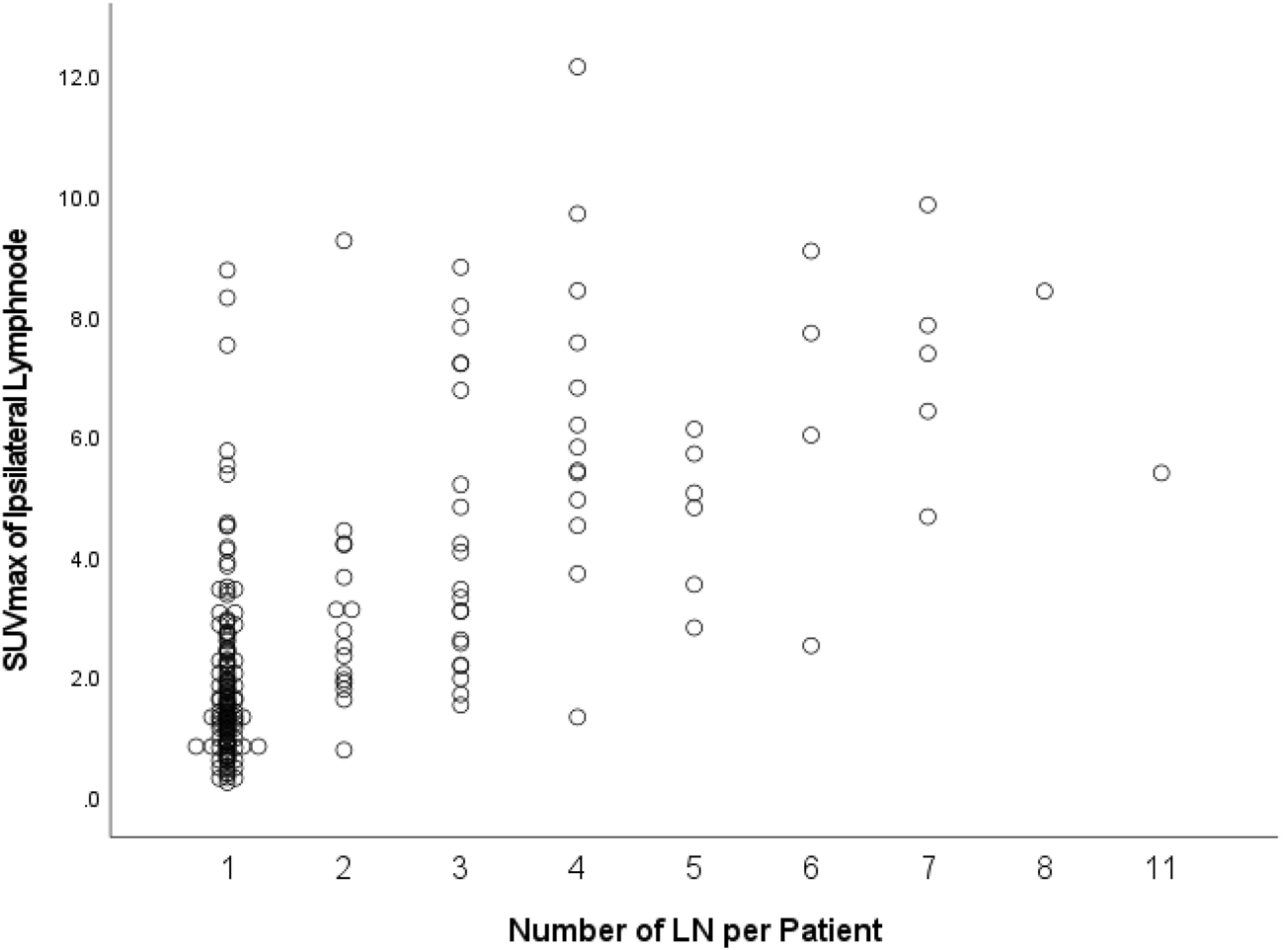
Correlation between SUVmax values and the number of detected lymph nodes.

More than one lymph node^2–11^ was detected in 67 patients (30.5%). We found a strong correlation between SUVmax values and the number of detected lymph nodes (r = 0.63, *p* < 0.001) (Fig. 2).

Axillary lymph node SUVmax had lower tracer activity in comparison to liver, bone marrow and spleen (Table 3).

**Table 3:** *p*-value of Wilcoxon signed-rank test for the difference against SUVmax of lymph nodes.

Region	Mean (SD)	Median (IQR)	*p*
SUVmax LN	2.76 (2.304)	1.90 (1.20–3.51)	
SUVmax liver	3.26 (0.768)	3.20 (2.75–3.75)	< 0.001
SUVmax mediast	2.55 (0.641)	2.45 (2.14–2.92)	0.229
SUVmax spleen	2.71 (0.782)	2.64 (2.30–3.14)	0.044
SUVmax bone Marrow	3.02 (1.749)	2.80 (2.26–3.48)	0.001

The most significant difference was the decrement of SUVmax in lymph nodes in relation to SUVmax in the liver (*p* < 0.001). This difference was also statistically significant in relation to spleen and bone marrow, with *p* values of 0.044 and 0.001, respectively (Table 3).

The time of vaccination correlates significantly negative with the tracer activity changes in axillary lymph nodes (r = − 0.26, *p* < 0.001).

In our research, we found that as patients age, there is a tendency for the SUVmax to decrease. Specially, the Spearman’s rank correlation coefficient (r) was calculated to be − 0.21, with a statistically significant *p*-value of 0.002. This correlation coefficient indicates a moderate inverse relationship between age and SUVmax, implying that as patients get older, the SUVmax values tend to be lower.

### Results in patients with pre-vaccination PET/CT examination

In this patient population, five patients (7%) were vaccinated with AstraZeneca, 36 (50.7%) with Moderna, and 30 (42.3%) with BioNTech/Pfizer, respectively. SUVmax of lymph nodes changed significantly after vaccination (*p* < 0.001) (Table 4). A significant change of tracer activity in the liver was also observed (*p* = 0.032). There was no significant change of tracer activity in other reference regions (mediastinum, spleen, and bone marrow).

**Table 4:** *p*-value of exact Wilcoxon signed-rank test (before vs after COVID-vaccination).

	Before or after vaccination	Mean (SD)	Median (IQR)	*p*
SUVmax of				
LN	Before	1.21 (1.233)	0.82 (0.59–1.38)	< 0.001
	After	2.75 (2.361)	1.80 (1.07–3.89)	
Liver	Before	3.49 (.992)	3.37 (2.83–3.91)	0.032
	After	3.17 (.821)	3.11 (2.56–3.70)	
Mediastinum	Before	2.62 (.700)	2.48 (2.15–2.94)	0.274
	After	2.50 (0.582)	2.46 (2.20–2.72)	
Spleen	Before	2.80 (0.964)	2.74 (2.23–3.48)	0.065
	After	2.62 (0.975)	2.71 (2.21–3.19)	
Bone Marrow	Before	2.81 (1.151)	2.58 (1.97–3.39)	0.460
	After	3.19 (2.796)	2.66 (2.17–3.56)	
SUVmax ratio of				
LN/Liver	Before	0.34 (.253)	0.25 (0.18–0.40)	< 0.001
	After	0.93 (0.895)	0.59 (0.39–1.20)	
LN/mediastinum	Before	0.46 (0.361)	0.32 (0.22–0.56)	< 0.001
	After	1.13 (0.992)	0.75 (0.48–1.58)	
LN/spleen	Before	0.56 (1.029)	0.31 (0.23–0.55)	< 0.001
	After	2.44 (7.158)	0.79 (0.45–1.69)	
LN/bone marrow	Before	0.45 (0.393)	0.32 (0.25–0.58)	< 0.001
	After	1.01 (0.961)	0.69 (0.36–1.39)	

The change in SUVmax ratio of tracer accumulation in lymph nodes compared to other regions was statistically significant (*p* < 0.001).

## Discussion

Rapid and word-wide vaccination programs with different types of vaccines were started shortly after the widespread outbreak of the ongoing global pandemic. As the immunization programs progressed, metabolically active axillary lymph nodes became apparent in the 2-[18F] FDG PET, increasingly facing a challenge for nuclear medicine physicians foremost in oncologic patients, as 2-[18F] FDG PET/CT may also be used to evaluate inflammatory and infectious diseases with good diagnostic accuracy^16–20^.

It is well-known from previous studies in other different vaccinations such as H1N1, influenza, hepatitis B that increased glucose activity at the injection site and ipsilateral lymph nodes caused by transient inflammation through macrophage accumulation. Eifer et al. reported 30% lymph node activity in 2-[18F] FDG PET/CT after H1N1 vaccination, whereas this activity was shown to be much higher after COVID-19 vaccination^21^. This dilemma in case of COVID-19 vaccination was first addressed in breast cancer imaging, where lymphadenopathy ipsilateral to the injection site after vaccination was reported^22^. Recently we observed a burst of publications regarding ipsilateral lymphadenopathy after vaccination against COVID-19.

The importance of this issue rises every day as we observe more vaccinated patients undergo PET/CT with different tracers. In recent studies and reports, hypermetabolic lymphadenopathy was also detected with other tracers^23–25^. Publications and reports with positive results are more likely to be published. Therefore, we have designed our study based on the tracer activity whether pathological or not. There are also numerous publications on ipsilateral lymphadenopathy because of immune response after administration of other traditional vaccines. This knowledge is therefore not new in the field of Nuclear Medicine.

However; in our study, we intended to present not only the ipsilateral lymph node activity, but also the tracer activity in liver, spleen and bone marrow after COVID-19 vaccination with different vaccines. We observed changes in lymph node activity and time interval after vaccination with three different vaccines (BioNTech/Pfizer, Moderna, and AstraZeneca). Recent studies report ipsilateral hypermetabolic lymph nodes in PET/CT in patients 1 day up to 4 weeks after vaccination. Dan et al. suggested in their study performed on patients vaccinated with BioNTech/Pfizer three “time windows.” They observed the lowest lymph node activity in the first 5 days and 3 weeks after the first dose of vaccine and at least 3 weeks after the booster dose^26^. Seban et al. reported a case in which a patient 5 days after vaccination with COVID-19 viral vector vaccine without any history of treatment or diseases such as chronic inflammatory showed high tracer activity not only in axillary lymph nodes but also in the spleen^27^. Shah et al. suggested a persistent 2-[18F] FDG activity up to 4 weeks after injection^28^. This approves our data to some extent. We also showed higher activity in the liver after vaccination in patients with the pre-vaccine examination.

In our study, lymph node activity after BioNTech/Pfizer and its relation to reference organs is at the highest at the 10th day with a gradual decrease over time. Almost 70 days after vaccination, the metabolism in the lymph node is not different from background activity. However, in the other two groups, we observed the highest peak of activity in the 4th week for Moderna and at the 10th day for AstraZeneca.

Our study showed no significant differences in tracer activity between three different vaccines, although there is a difference in vaccine components. It has yet to be studied in the future. The pattern of response and changes in immune cells at the site of injection can be evaluated for vaccine efficacy and changes of this pattern during the time^29^.

**Figure 3:**
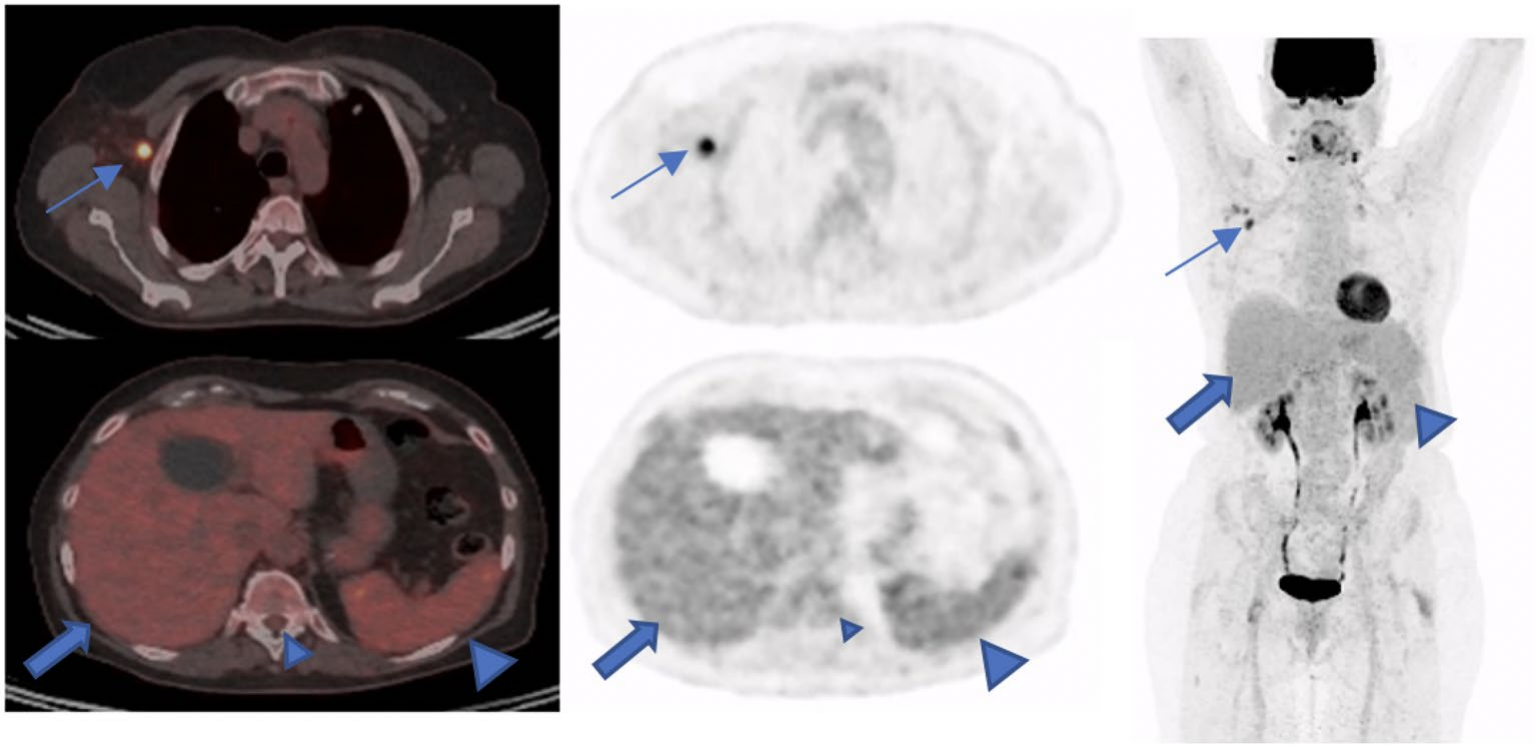
A 73-old woman with a suspicious lung nodule was referred to our division for F18-FDG PET/CT examination. She was vaccinated 4 days before PET/CT. The lung nodule did not show any pathological FDG-Uptake; however, high tracer activity was detected on her right arm, where she was injected, and multiple hypermetabolic lymph nodes in the right axilla (arrows). Moreover, PET/CT showed high tracer activity in the liver (black arrows), spleen (triangular arrows), and bone marrow (small triangular arrows) as the result of systemic immune response after vaccination. The tracer defect in the liver was due to the known cyst. (**A**) fusion scan, (**B**) PET, (**C**) maximum intensity projection.

Our study also showed the metabolic changes in lymph nodes and reference regions in a group of patients with pre-vaccination PET/CT examinations. In this patient population, we observed a significant change of metabolic activity in axillary lymph nodes and the liver. It shows the importance of knowledge of physiological and/or pathological changes of immune response after vaccination in the field of Nuclear Medicine. It is also shown in other studies that diffuse 2-[18F] FDG-activity can be seen in the spleen as a result of immune response after COVID-vaccination^30^ Figure 3.

It is an attempt to minimize the dilemma, especially in oncologic diagnostics. Furthermore, a more accurate staging leads to better therapy management in different diseases. It mainly plays an essential role in patients with lymphoma.

Response evaluation with Deauville-Score system in PET/CT based on tracer activity in lesions, liver, and mediastinum for different lymphomas has been well established^31^. This may also be affected due to tracer activity changes in the liver. Furthermore, the therapy regime and related side effects can be a hurdle for patients and the healthcare system because of false evaluation. It has to be further investigated in different trials. Our study is also a preliminary attempt to show this influence.

### Limitation of the study

While this study provides valuable insights into the metabolic activity changes following COVID-19 vaccination using different vaccines, several limitations should be acknowledged. First, the inclusion of patients with varying clinical states such as those with fever of unknown origin (FUO) or liver diseases introduces potential confounders that might impact the results. Patients with underlying medical conditions can exhibit alteration in glucose metabolism and immune response which could affect the interpretation of a 2-[18F] FDG -PET/CT findings. Additionally, the presence of comorbidities like liver diseases can influence hepatic tracer uptake and metabolism, potentially leading to variation in reference organ values. Furthermore, the influence of medication particularly in patients with autoimmune conditions like rheumatoid disorders may also contribute to variation in FDG consumption of reference organs. Medications use to manage these conditions such as corticosteroids or immune suppressants, can modulate glucose metabolism and affect the standardized uptake values of reference tissues. Consequently, the study’s findings may be subject to confounding factors related to patients’ clinical backgrounds and medication regimes. The study’s relatively small and homogeneous sample size, primarily consisting of the Caucasian population of Austria, further limits the generalizability of the results. The retrospective nature of the study also presents inherent biases making it challenging to control for all potential confounding variables. Additionally, the variability in the time interval between pre-vaccination and post-vaccination PET/CT examinations within the subgroup of patients who underwent both scans should be considered when interpreting these results.

In summary, while this study offers valuable insights into the metabolic changes observed in various tissues following COVID-19 vaccination, it is important to acknowledge the limitations associated with the inclusion of patients with different clinical states and their potential impact of medication and reference organ SUVs. These factors can introduce confounding elements that should be considered when interpreting the study’s findings. Furthermore, the limited sample size and the retrospective design called for cautious generalization of the results to broader patient populations.

SUVmax. is a valuable metric in assessing metabolic activity in PET imaging but it is susceptible to partial volume effects particularly in small structure like lymph nodes. Researchers and clinicians need to be aware of these limitations when interpreting PET/CT scans and consider alternative metrics like SUVpeak to improve consistency and minimize the risk of false interpretation especially in patients with oncological conditions.

SUVpeak is as a potential improvement, indicating reduced image noise and bias^32^.

## Conclusion

In conclusion numerous studies suggest increased activity in local sites and ipsilateral axillary lymph nodes in 2-[18F] FDG PET/CT scans after COVID-19 vaccination. However; correctly interpreting these changes remains a challenge particularly in patients with oncological diagnoses. The type of vaccine administered and the time interval between vaccination and PET/CT scans are critical factors in minimizing false interpretations, particularly in the case of therapy evaluation. our study underscores the significance of changes in 2-[18F] FDG PET/CT scans in lymph nodes and reference organs after vaccination and highlights the importance of this information in the interpretation of PET/CT in vaccinated patients, particularly in lymphoma, where the Deauville-Score plays a crucial role in therapy management.

## Data Availability

The datasets generated and/or analyzed during the current study are not publicly available due to confidentiality concerns but are available from the corresponding author on reasonable request at any time.
